# Feasibility of Recruiting Psychiatrically Hospitalized Adults for a Randomized Controlled Trial of an Animal-Assisted Intervention

**DOI:** 10.3390/healthcare14020154

**Published:** 2026-01-07

**Authors:** Lisa Townsend, Nancy R. Gee, Erika Friedmann, Megan K. Mueller, Tushar P. Thakre, Sandra B. Barker

**Affiliations:** 1 Department of Pediatrics, School of Medicine, Virginia Commonwealth University, Richmond, VA 23284, USA; 2 Center for Human-Animal Interaction, Department of Psychiatry, School of Medicine, Virginia Commonwealth University, Richmond, VA 23219‌, USA; nancy.gee@vcuhealth.org (N.R.G.); sbbarker@vcu.edu (S.B.B.); 3 Department of Organizational Systems and Adult Health, University of Maryland School of Nursing, Baltimore, MD 21201, USA; 4 Department of Clinical Sciences, Cummings School of Veterinary Medicine, Tufts University, North Grafton, MA 23298‌, USA; megan.mueller@tufts.edu; 5 Department of Psychiatry, School of Medicine, Virginia Commonwealth University, Richmond, VA 23284, USA

**Keywords:** animal-assisted intervention, loneliness, depression, psychiatry, acute care

## Abstract

**Background/Objectives**: Evaluating the feasibility of randomized controlled trials (RCTs) represents a critical next step for advancing human–animal interaction (HAI) science and rigorously exploring the role of animal-assisted interventions (AAIs) in psychiatric acute care. This study presents strategies for conducting a pilot RCT comparing an animal-assisted intervention involving dogs (AAI) with an active conversational control (CC), which incorporated conversation with a human volunteer, and treatment as usual (TU) for improving mental health outcomes in psychiatrically hospitalized adults. **Methods**: We recruited participants from an acute-care psychiatric unit at an academic medical center. AAI and CC were delivered by volunteer handlers with and without their registered therapy dogs. Feasibility data included number of recruitment contacts, recruitment rate, and reasons for non-enrollment. We describe recruitment challenges encountered and mitigating strategies for successful study completion. **Results**: Recruitment occurred over 23 months with a goal of 60 participants participating in at least one intervention day. A total of 264 patients were referred to the study and 72 enrolled. The additional 12 people were recruited to replace participants who did not complete any intervention days and did not provide any intervention data. Study recruitment goals were met with a recruitment rate of 27.30%. **Conclusions**: Research to improve the lives of patients in acute psychiatric care is a vital public health goal, yet RCTs are difficult to conduct in acute care settings. Studies like this strengthen HAI and psychiatric science by providing a roadmap for implementing successful AAI RCT designs.

## 1. Introduction

Although RCTs are costly and challenging to conduct, they offer benefits that are not possible with other designs [1]. Most importantly, RCTs minimize threats to internal validity, which, in intervention research, is the inference that the intervention causes the hoped-for change in outcomes [2]. Confidence in conclusions drawn regarding cause-and-effect relationships is critical when evaluating healthcare interventions so that useful treatments are not discarded due to spurious negative results or adopted following specious positive ones. RCTs conducted with psychiatrically hospitalized populations face the challenge of recruiting patients who are highly symptomatic, dealing with multiple life challenges, and undergoing intense treatment for crisis stabilization. Nevertheless, such trials are vital for improving knowledge regarding the best treatments and engagement strategies for adults in acute psychiatric settings.

Psychiatric inpatient care is characterized by high patient need, a busy environment, and increasingly short lengths of stay. People whose mental illnesses require hospitalization often enter their inpatient stays in crisis [3], facing significant symptoms and social consequences that compound their distress [4,5]. Patients indicate that they want inclusivity, positive patient/provider relationships, a therapeutic environment, and a home-like atmosphere [6]. Surveys regarding inpatient experiences often reflect the tension between staff’s charge to achieve therapeutic and pragmatic goals and patients’ need to feel heard and seen [7], as well as receive individualized care [8]. Successful delivery of inpatient therapies must account for the emotional and cognitive challenges facing those in crisis and create a welcoming, safe space that invites patients to engage [9].

The complexity of treating a milieu of patients in crisis can limit the time staff have to provide individualized treatment in a soothing atmosphere. Many clinical goals must be achieved in a short time, including psychiatric stabilization, medication adjustments, and securing post-discharge resources. Characteristics of patients’ illnesses, such as psychosis or externalized anger may strain provider resources, create stress and fear in the milieu, and inhibit therapeutic engagement [10].

Adjunctive treatments may attenuate these challenges by easing stress and engaging patients while simultaneously alleviating staff burden. A variety of interventions have been explored, including yoga [11], high intensity interval training (HIIT) [12], sleep coaching [13], acceptance and commitment therapy [14,15,16], mindfulness-based crisis intervention [17], and brief cognitive behavior modules [18,19,20]. Few RCTs have examined animal-assisted interventions (AAIs) with therapy dogs as adjunctive acute care therapies.

Therapy animals have played a role in treating mental health symptoms for decades [21] and are commonly involved with outpatient psychotherapies to facilitate patient engagement and skill development [22]. Although previous research suggests that working with a therapy animal confers positive mental health benefits for psychiatric inpatients [23,24,25], studies of human–animal interaction (HAI) have only recently begun to establish an evidence base incorporating RCT methodology. Kamioka and colleagues [26] summarize several randomized trials involving a variety of therapy animals from dogs and cats to birds, farm animals, and even dolphins. Therapies ranged from short interactions in the hospital (therapeutic visits with patients or residential small animals on inpatient units) to interacting with animals in their habitats (caring for farm animals or snorkeling with dolphins.). Kamioka et al. [26] conclude that interventions involving animals show promise for improving a variety of health and mental health outcomes for patients who like animals. The authors highlight that greater standardization of methods and reproducibility of intervention protocols will improve the quality of information provided by RCTs.

RCT designs provide more robust information regarding efficacy than other methods and establish scaffolding for studying mechanisms of effect. The RCT framework supports inferences regarding whether interventions are efficacious and allow further mechanistic analyses that speak to why a treatment succeeds or fails [27]. HAI and psychiatric science stand to benefit from elucidation of patient populations that can benefit from AAIs, appropriate timing and dosing of these interventions, and the mechanisms by which human–animal interactions catalyze their effects [28,29,30]. An important step toward building an evidence base for AAIs with dogs as adjunctive therapy is to determine the feasibility of enrolling psychiatry inpatients in randomized controlled studies of these interventions.

Most RCTs with psychiatric inpatients have evaluated brief psychotherapies [11,12,13,14,15,16,17,18,19,20,31]; few have examined whether adjunctive AAIs facilitate symptom reduction. This paper describes recruitment for a pilot feasibility randomized controlled trial; the benefits of these reduced scale RCTs are to ensure that participants are willing to be in the study, that the intervention can be delivered as intended, and that a signal can be detected from the measures employed. Pilot feasibility designs allow improvements to be made before significant investment of time, money, and effort are made on a larger trial [32]. In addition, our study strengthens the HAI and psychiatric literature by employing a design that isolates the differential contributions of the role played by the human handler and the contributions of the therapy dog and addresses standardization concerns by controlling session timing, delivery, and dose. This paper focuses on recruitment feasibility. We present study design features that facilitated recruitment along with adaptations to enhance implementation of future trials. Findings are presented according to CONSORT guidelines for pilot feasibility trials [33].

## 2. Materials and Methods

### 2.1. Design

The study was reviewed and approved by the university Institutional Review Board (IRB) and underwent ethical review by a human subjects ethics committee in state government that oversees research with psychiatric inpatients. It was exempted by the university’s Institutional Animal Care and Use Committee. It was a pilot feasibility RCT with enrollment as a primary outcome. The study aim for this paper examines the feasibility of recruiting psychiatrically hospitalized patients for an AAI RCT. The aims of the RCT itself were to (1) evaluate the feasibility of a therapy dog visitation intervention (AAI arm) over three consecutive days compared to a conversational control condition with the handler alone (CC) and a treatment-as-usual control (TU); (2) assess the efficacy of AAI compared to CC and TU for improving depression, anxiety, mood, loneliness, and health-related quality of life outcomes; and (3) obtain data to estimate sample size for a larger RCT. This paper focuses on recruitment feasibility and describes the full trial to provide context for evaluating recruitment results. Results of the completed trial are available elsewhere [34].

### 2.2. Intervention

The dogs were privately owned, registered therapy dogs in the Center for Human–Animal Interaction “Dogs on Call” (DOC) program [35]. DOC is an evidence-based program that provides therapy dog visits at an academic medical center. All handler-dog teams must be registered with Pet Partners or Alliance of Therapy Dogs with an American Kennel Club Canine Good Citizen title. Additionally, DOC teams undergo on-site evaluations to ensure that the dogs are comfortable in the hospital setting. The handlers are hospital volunteers who undergo health screening, fingerprinting, background checks, flu vaccinations, and trainings on patient interactions and confidentiality. Handlers receive ongoing education regarding canine consent, recognizing stress and fatigue in their therapy dogs, and smoothly ending a visit if their dogs exhibit tension or fatigue. The intervention phase of the study required five days, including baseline, three intervention days, and post-intervention measures.

The interventions in the three study arms are described below. Twenty participants were randomly allocated to each condition. The enrollment goal of 20 participants per condition was based on a priori power analyses [36].

(1) AAI: Participants took part in a 20 min, non-scripted interaction with a DOC therapy dog and handler team on three consecutive days. The handlers were provided with specific general conversation topics to facilitate intervention fidelity and consistency.
(2) CC: Participants interacted with a therapy dog handler who visited them without a dog and conducted a 20 min, informal social interaction as described above. The sessions were repeated on three consecutive days.
(3) TU: Participants received treatment as usual without any additional intervention. They engaged in their usual activities, such as watching television during the 20 min “intervention” period.

The rationale for the 20 min intervention length was to closely approximate the length of the therapy dogs’ regular hospital visits; this provided valuable information regarding whether the intervention as it naturally occurred in the hospital visitation program was associated with improved patient outcomes. Participants in the control conditions were not allowed to visit with the dogs during the study, as that would have contaminated the treatment conditions and the associated assessments as well as and the follow-up measures. During the informed consent process, participants were informed that they had a 33% chance of being randomly allocated to the dog condition and a 66% percent chance of being assigned to the control conditions. Participants were informed that they had the option of withdrawing from the study if they did not like the condition to which they were assigned.

Participants were asked to complete a set of 1- and 6-month follow-up measures following their hospital discharge. We chose these timepoints to examine the sustainability of intervention effects. All participants received standard treatment, including crisis stabilization, medication management, group therapies, and discharge planning.

**Figure 1:**
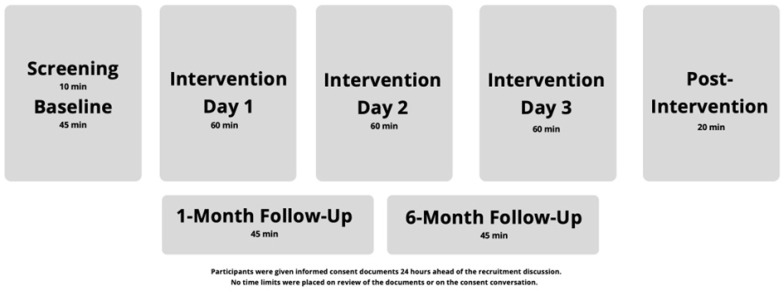
Study Structure and Timing.

Figure 1 illustrates the study structure and the timing and length of data collection and intervention visits.

### 2.3. Participants

Participants were adults hospitalized due to exacerbation of mental health symptoms. Length of stay for most patients was approximately one week. The study was trans-diagnostic, including patients with depressive and anxiety disorders as well as primary psychotic illnesses if participants were able to provide informed consent. Table 1 provides the study inclusion and exclusion criteria.

### 2.4. Measures

*Brief Interview for Mental Status (BIMS).* The BIMS [37] measures cognitive functioning and was used to screen for cognitive or attentional issues that could impair a person’s ability to provide valid informed consent. Participants were eligible for study participation if they received a score of 13 or greater.

*Demographic Information*. This includes age, gender, marital status, race, ethnicity, educational attainment, and employment status.

*Pet Ownership*. This included the number and type of current and past companion animals.

*UCLA Loneliness Scale, Long and Short Forms (UCLA-LS/UCLA-SF).* This scale measures subjective experiences of loneliness and social isolation [38,39]. The long form (20 items) was used at Baseline, Post-Intervention, 1- and 6-month follow-ups. The short form (6 items) was used before and after the intervention visit on the three intervention days.

*Analog Loneliness Scale.* This single-item measure was used to measure state loneliness before and after the intervention visit on the three intervention days [40].

### 2.5. Setting

The study site was an adult inpatient psychiatry unit at an academic medical center. This unit served general psychiatry inpatients whose symptoms did not pose significant risk of aggressive behavior and did not preclude understanding of study information or provision of informed consent.

### 2.6. Recruitment Procedures: Participant Identification and Consent

All clinical staff were informed about the study and given information packets for patients. The study team visited the unit at the start of each week to obtain a list of patients they were approved by staff to approach. Clinical staff referred patients unless study participation was contraindicated in their clinical judgment. Examples of such contraindications included patients who felt overwhelmed by their hospitalization or environmental stressors or exhibited psychotic symptoms that impacted their ability to provide informed consent. Patients were referred to the study team within 24 h of admission. Recruiters spent time on the unit daily working through the referral list and checking with unit staff for new referrals. The study team approached patients in their rooms or in the general milieu after seeking permission from clinical staff. Interested patients were given an information packet that outlined study procedures and contained informed consent documents for review. Potential participants were approached a maximum of three times unless they provided a clear indication before then that they did not wish to continue discussing the study. In that case, the study team discontinued contact attempts. Participants who wished to continue talking about study participation but were unavailable or undecided for three contact attempts were re-contacted beyond the three attempts. This allowed us to recruit people who remained interested but whose schedules made it difficult to meet with the recruitment team. We also looked for and honored passive non-consent, which was represented by non-verbal communication, body language, or other subtle indications that the person did not wish to talk with the study team. Participants were informed that they could withdraw from the study at any time. These strategies allowed recruiters to avoid pressuring people to enroll while maximizing the ability to recruit those patients whose treatment regimens required multiple contacts before recruitment could be completed.

Prior to, and during this trial, the study team developed several strategies that can inform similar RCTs, with specific recommendations for researchers investigating AAI’s in this population.

### 2.7. Laying the Groundwork

#### 2.7.1. Strong Relationship with Clinical Staff

An established relationship with unit staff formed an important foundation for the study’s success. The DOC program was established in 2001 by a psychiatry faculty member (a member of this study team). DOC provides therapy dog visits to staff, families, inpatients, and ambulatory care patients in most areas of an academic teaching hospital. DOC forms a vital part of a center that focuses on HAI research, education, and practice, much of which has been performed in collaboration with psychiatry faculty. One of the study collaborators serves as Medical Director of the adult psychiatry inpatient unit. Several members of the study team have provided clinical and DOC services and conducted research in collaboration with inpatient unit staff—thus, the study team and DOC program were respected by the staff providing participant referrals.

#### 2.7.2. Program Popularity

The DOC program is well-known for comforting patients and families throughout the health system. As such, patients who receive services at the medical center are often familiar with DOC, either by previous experience or reputation. DOC dogs have their own “trading cards” featuring their name and photo which patients and staff often collect. DOC also has a social media presence, which contributes to the program’s acceptance and familiarity among those who use hospital services. The therapy dogs themselves were a specific draw for patients who loved animals or had a particularly soft spot for dogs. Anecdotally, many patients visibly brightened at the idea of receiving a visit from a therapy dog during their hospital stay. The strong relationships with clinical staff and the program’s popularity paved the way for providers and patients to welcome study recruitment efforts.

#### 2.7.3. Recruitment Uptake Outcomes

Study referrals and recruitment outcomes were tracked, including patient name, contact date, and outcome. Outcome codes were categorized as follows: (1) enrolled; (2) declined; (3) considering participation; (4) screen failure; (5) patient unavailable; (6) not appropriate for the study; (7) missed opportunity; (8) duplicate referral. “Considering participation” indicated patients who remained undecided about whether to join the study. “Screen failures” represented patients who expressed interest in the study but did not meet inclusion/exclusion criteria. “Patient unavailable” was used for patients who could not be contacted for a variety of reasons, including being asleep, attending medical/therapeutic procedures/sessions, or being discharged before contact could be made. The “not appropriate for the study” code included patients who were transferred elsewhere or were scheduled to discharge before study procedures could be completed. “Missed opportunities” included patients who expressed interest in the study but were not re-contacted due to study team time constraints. “Duplicate referral” codes were used for patients who were re-admitted to the unit after having been contacted previously or who had already enrolled and completed study procedures. If participants spontaneously provided a reason why they did not wish to participate, the reason was recorded.

An overall recruitment rate was calculated and defined as the number of consented participants divided by the number of potential participants referred to the study by clinical staff. The a priori criterion for deeming recruitment to be successful was the ability to recruit 60 participants (20 per study arm), based on a priori power analyses [36]. This paper presents recruitment findings and places them in the context of recruitment for other pilot feasibility trials conducted on inpatient psychiatry units. The comparison studies were identified through a literature search based on population (adults with serious mental illness), setting (inpatient psychiatry), evaluation of an intervention feasibility RCT, and reported or calculable ratio of consented vs. potential participants.

## 3. Results

**Figure 2:**
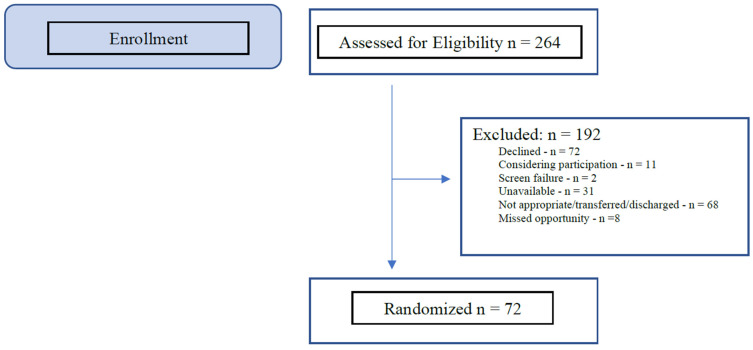
Modified CONSORT Enrollment Flowchart-Recruitment Outcomes.

Recruitment began in March 2022 and ended in January 2024 (23 months). A total of 264 patients were referred to the study, of which 72 enrolled. Figure 2 presents a modified pilot feasibility CONSORT chart depicting recruitment outcomes. The full CONSORT chart is presented elsewhere [34]. Twelve additional participants were enrolled because twelve previously enrolled participants were unable to complete any of the intervention days so they were replaced to attain 60 participants with usable intervention data. Nine of the participants who withdrew before participating in the intervention changed their mind about study participation. Only one of these withdrew because they did not like their assigned treatment condition (TU).

Study recruitment goals were met. The overall recruitment rate was 27.30%. The average number of contacts made to potential participants was 1.97 (SD = 0.91), range 1 to 5. The “considering participation” category in the CONSORT figure represents potential participants who expressed the wish to continue talking with recruiters about the study, but who remained undecided. In those instances, recruiters continued to engage and answer questions about the study. Given the relatively short length of stay on the acute care unit, this often meant that discharge dates approached for these undecided patients, which ultimately made them ineligible for the study because they would not be able to receive the full intervention. These data were included in the denominator for enrollment rate calculation and lowered the recruitment rate.

Table 2 provides demographic information for the 72 patients who enrolled in the study. This information was not gathered for those who declined participation. The sample was almost evenly divided between men and women, with a small number of gender non-binary participants. Most of the sample identified as white. Many were single and unemployed, which is consistent with the social demographics often found for adults with serious mental illness [5,41,42]. Nearly everyone in the sample reported having at least a high school or equivalent education, with half of the participants reporting attending college.

Table 3 provides recruitment rates for similar feasibility RCTs conducted on inpatient psychiatry units in the US, Europe, and Japan. Our recruitment rate (27.30%) falls at the lower end of rates reported by these studies, which ranged from 16.56% to 96.61%.

Next, we present recruitment challenges specific to our study along with strategies we used to mitigate them.

### 3.1. Recruitment Challenges and Mitigating Strategies

#### 3.1.1. COVID-19

Recruitment began in March 2022, approximately two years after the onset of the COVID-19 pandemic. During the initial outbreak, the DOC program was placed on hiatus until understanding of contagion sources was developed. DOC resumed hospital visitation approximately one year after the initial COVID-19 outbreak [44] in accordance with Centers for Disease Control [45] and American Veterinary Medical Association [46] protocols. However, viral mutations, the time-consuming nature of public vaccine roll-out, and the communal milieu of the psychiatry unit caused repeated unit closures when inpatients tested positive for SARS-CoV-2. When this occurred, the unit closed to visitors, diverted patient intakes, and implemented isolation protocols, which resulted in temporary recruitment suspension. Each unit closure lasted for approximately 5–10 days. Given that public roll-out of the COVID-19 vaccine was in early stages, the study team encountered multiple unit closures during the study.

#### 3.1.2. Mitigating Strategy: Communication with Sponsor

The COVID-19 pandemic brought about unprecedented challenges to intervention research. Our study did not have a virtual option and depended upon in-person participant interactions. Although we could not alter the timing and duration of unit closures, we communicated consistently with our study sponsor regarding recruitment challenges and with psychiatry unit staff to extend our timeline and resume enrollment efficiently following each unit closure.

### 3.2. Early Discharge, Intensive Treatment

Inpatient stays are notoriously short in the United States; our study recruitment was hampered by patients’ ultra-brief stays combined with busy treatment schedules as they received crisis stabilization and participated in discharge planning. The study team attempted to meet with referred patients directly after receiving referrals from staff. Out of respect for patients’ recovery, the team did not awaken sleeping patients, disrupt treatment services, or interrupt visits from family or friends to talk with patients about the study. The team approached patients during their free time. This built rapport with staff and patients, as it highlighted the importance the study team placed on recovery; however, it was time- and effort-intensive for study recruiters.

**Mitigating Strategy: Flexible Study Team Staffing and Hours.** The study team allocated several staff members to ensure adequate unit coverage and scheduled recruiters flexibly given that multiple attempts were often needed to deliver study information and conduct informed consent. Documentation of each contact attempt was recorded and coordinated by a faculty member who oversaw recruitment. This allowed timely information to be passed on to the next recruiter. Study staff schedules accommodated weekend recruitment.

**Mitigating Strategy: Intent to Treat (ITT) Data Analytic Methods.** Given that one of the RCT’s primary goals was to assess intervention feasibility, the study team accounted for potential missed visits in creating the data analysis plan. We considered a participant’s data usable if they completed at least one intervention visit and employed intent to treat analysis strategies. This allowed us to use data if a patient discharged unexpectedly and to complete recruitment efficiently without replacing participants who discharged before completing all three intervention visits. Although this mitigation applies to the RCT’s intervention outcomes [34] we describe it here because planning this data analysis strategy prior to recruitment facilitated our ability to complete the study more efficiently.

### 3.3. Working with a Volunteer Research Workforce

Our intervention was delivered by registered volunteer therapy-dog handlers and therefore depended upon their availability. Given that enrollments occurred throughout the week at varied times, teams often had only 24 h notice of a scheduled intervention visit. Intervention visit times could not be changed easily because patient schedules were relatively inflexible. Additionally, DOC volunteer handlers often worked full- or part-time at other jobs and did not have set volunteer schedules.

**Mitigating Strategy: Volunteer Capacity-Building.** Involving a volunteer workforce in an RCT poses a risk that handler-dog teams may be unavailable when patients are free to participate in the intervention. The study team compensated for this by recruiting and training a total of 19 teams and developing a streamlined, IRB-approved human subjects training adapted for the study [47]. The university’s IRB designated the volunteer handlers as “community-engaged researchers” because they delivered the intervention to study participants. Thus, the teams were required to undergo human subjects research protections training before conducting research activities. We collaborated with the IRB to create an hour-long, interactive, bespoke training that reviewed human subjects research considerations specific to our study. This tailored training required substantially less time for volunteers to complete than traditional CITI trainings and allowed us to onboard enough teams to cover intervention visits on weekdays and weekends. Furthermore, we maintained a loose schedule of volunteer availability that structured which teams were contacted on specific days and times to maximize visit coverage. Of note, all 60 Dogs on Call teams were invited to join the research project and receive the training; 19 teams responded, which represents a 33% response rate.

## 4. Discussion

We gathered usable data from 60 adults on an inpatient, acute care psychiatric unit, successfully meeting our recruitment goal (20 per arm) with an overall recruitment rate of 27.30%. Twelve participants were replaced because they either withdrew or unexpectedly discharged before providing any intervention data. We successfully navigated many challenges reported with other RCTs in acute care psychiatric settings, including busy patient schedules, unexpected early discharges, and abbreviated lengths of stay. Below, we place our findings in the context of other RCTs in acute care psychiatry and discuss additional adaptations that could enhance recruitment in future studies.

### 4.1. Contextualizing Our Findings

Other RCTs evaluating interventions in acute care psychiatric settings have met with varying degrees of recruitment success. An intervention intended specifically for patients with psychosis [17] demonstrated the lowest recruitment rates (16.56%), likely due to the severity of impairment faced by these patients, followed by an exercise intervention [12] at 38.16%. Most interventions in the comparison group studied abbreviated versions of evidence-based psychotherapies adapted for acute care, such as acceptance and commitment therapy (ACT), mindfulness, and cognitive behavioral therapy delivered adjunctively to standard treatments. Some trials were conducted in psychiatric units with longer lengths of stay or in non-U.S. countries where inpatient programming might differ from that provided in the U.S.; these trials demonstrated the highest recruitment rates among the comparison studies. Geographic location and associated policies regarding inpatient treatment and length of stay may influence the success of RCTs. Studies with higher recruitment rates have been conducted in areas of the world characterized by much longer inpatient stays [11], allowing more time for research interventions to be completed. Although our study had one of the lower recruitment rates compared to other trials, we achieved our recruitment goals. Several characteristics of our approach likely contributed to this success—we review these below and include additional adaptations to enhance recruitment in future trials.

Our study benefited from the widespread appeal of therapy dogs to patients and providers—the program receives high patient satisfaction scores and the research study was enthusiastically welcomed by unit staff. The study visits were well-received with little demand upon patients. The greatest effort required from participants was completing mood and anxiety measures, which were similar to instruments they completed daily in the context of their clinical care. Thus, the cognitive load of participation was low. The study required a five-day intervention phase which was designed to fit within an average week-long inpatient stay. Evlat and colleagues [31] provide evidence for the need to accommodate busy patient schedules and short lengths of stay when implementing inpatient therapies. Our recruitment timeline could have been shortened with a briefer intervention model that required fewer days [20]. Additionally, capturing measures at discharge is complicated due to the number of activities that need to be accomplished then [15]. Our study offered flexibility regarding post-intervention measures, scheduling around discharge or leaving measures to be completed by paper and pencil before discharge activities commenced.

Flexible inclusion criteria and adapting interventions to patients’ busy timelines are needed to complete RCTs successfully in psychiatrically hospitalized populations [14]. Similarly, our study used broad, trans-diagnostic inclusion criteria and flexible study team availability to accommodate patient and unit schedules. Of note, the study team was available seven days a week, which is an important consideration when planning future studies of this nature. The study team also cultivated a strong communication protocol to increase the likelihood of patient referrals shortly after admission to accommodate the full intervention.

### 4.2. Implications for a Larger RCT

We conclude that recruitment is feasible for this type of AAI RCT. The COVID-19 pandemic posed the greatest barrier to study recruitment and extended the projected timeline for study completion. As vaccinations became available and public uptake increased, unit closures reduced. It is likely that in more typical, non-pandemic periods, recruitment will be more efficient. The study intervention was well-received by staff and participants with approximately 1 in 3 patients approached agreeing to participate. The appeal of AAIs sets a favorable stage for other RCTs of this kind.

Moving forward, we recommend employing at least two full-time research assistants whose time is dedicated to recruitment and data collection seven days per week. Future adaptations might consider streamlining the intervention to fit more comfortably within brief inpatient stays while retaining the flexibility that allowed us to pivot and adapt to changing patient schedules.

## 5. Conclusions

Engaging psychiatric inpatients is a vital public health goal. AAIs often ease interpersonal interactions [28] and may engage patients in treatment while easing the distress that hampers stabilization. AAI studies such as this one strengthen HAI science by providing a roadmap for successful implementation of RCT methods and offering strategies for successfully implementing these much-needed research designs. This design also contributes to the knowledge base for psychiatric acute care by examining whether adjunctive AAI facilitates symptom reduction among inpatients. If findings support this type of intervention, AAI could be used to enhance patient engagement and promote stabilization while alleviating staff burden.

## Figures and Tables

**Table 1:** Study Inclusion/Exclusion Criteria.

**Inclusion Criteria**
English speaking
Age 18+
Projected hospital admission for 5 days
Access to phone after discharge
Ability to provide informed consent as measured by:
BIMS score ≥ 13
Not requiring guardianship
Clinical judgment of healthcare team
**Exclusion Criteria**
Allergy to or fear of dogs
COVID-19+
On contact precautions

**Table 2:** Demographic Data for Study Enrollees.

Variable	Category	n (%)
**Gender**		
	Woman	34 (47.2)
	Man	30 (41.7)
	Non-binary	7 (9.7)
	Missing	1 (1.4)
**Marital Status**		
	Single	37 (51.4)
	Married	20 (27.8)
	Divorced	11 (15.3)
	Widowed	3 (4.2)
	Missing	1 (1.4)
**Race**		
	White	42 (58.3)
	Black	22 (30.6)
	Asian	4 (5.6)
	American Indian/Alaskan Native	2 (2.8)
	Missing	2 (2.8)
**Hispanic/Latine Background**		
	No	65 (90.3)
	Yes	3 (4.2)
	Missing	4 (5.6)
**Education**		
	<High school	5 (6.9)
	High school/GED	20 (27.8)
	Some college	25 (34.7)
	Bachelor’s degree	13 (18.0)
	Graduate degree	9 (12.5)
**Employment**		
	Student	3 (4.2)
	Unemployed (not looking for work)	15 (20.8)
	Unemployed (looking for work)	20 (27.8)
	Self-employed	2 (2.8)
	Employed part-time	7 (9.7)
	Employed full-time	14 (19.4)
	Retired	8 (11.0)
	Missing	3 (4.2)

**Table 3:** Recruitment Rates for Comparison Acute Care Psychiatry Studies.

Author	Year	Intervention	Intervention Length	Site	Number of Admissions or Charts Reviewed	Potential Eligible	Number Screened or Approached	Number Consented	Number Randomized	Recruitment Rate (%) (Consented/Potential Eligible)
Ikai [11]	2017	Chair yoga vs. treatment as usual (TU)	2x weekly for 12 weeks	Japan		-----	64	60	56	-----
										
Gaudiano [16]	2023	Acceptance and Commitment Therapy (ACT)	50-min sessions	US	534	144	100	62	46	43.06
		vs. time and attention control	minimum of 3 individual and 3 group sessions							
			variable depending on length of stay							
										
Boden [14]	2016	ACT vs. TU	4 individual sessions	US Veterans hospital	429	67	67	29	18	43.28
										
Jacobsen [17]	2020	Mindfulness-based Crisis Intervention (BMCI)	1-5 individual sessions	UK	676	302	175	50	50	16.56
		vs. social activity control		patients with psychosis						
										
Martland [12]	2024	High Intensity Interval Training (HIIT)	2x weekly for 12 weeks	UK	-----	76	30	29	19	38.16
		vs. TU exercise at hospital gym								
										
Sharma [20]	2022	Cognitive Behavioral Therapy (CBT) app	free app access for 6 days	Canada	-----	33	28	24	20	72.73
		vs. TU	minimum use of 10 min daily							
										
Sheaves [13]	2018	CBT-based insomnia treatment	≥5 sessions over a 2-week window	UK	109	61	61	40	40	65.57
		vs. TU								
										
Westermann [43]	2021	Positive mental imagery vs. cognitive control	8 15-min sessions over 2 weeks	Germany	-----	59	59	57	57	96.61
		compared to TU								
										
Wood [6]	2018	CBT-based anti-stigma intervention	2 h over 2 weeks	UK	-----	45	45	30	30	66.67

Recruitment rates are not directly comparable because metrics were reported differently across studies. Standardization was attempted by calculating the number of participants consented/potential eligible when that information was available.

## Data Availability

This study was registered with ClinicalTrials.gov (Identifier: NCT05089201). The data for this report are included within the paper. Data from the full RCT can be accessed via reference [34].

## References

[1] Hariton E., Locascio J.L. (2018). Randomised controlled trials: The gold standard for effectiveness research. BJOG.

[2] Shadish W.R., Cook T.D., Campbell D.T. (2002). Statistical conclusion validity and internal validity. Experimental and Quasi-Experimental Designs for Generalized Causal Inference.

[3] Walker S., Mackay E., Barnett P., Rains L.S., Leverton M., Dalton-Locke C., Trevillion K., Lloyd-Evans B., Johnson S. (2019). Clinical and social factors associated with increased risk for involuntary psychiatric hospitalisation: A systematic review, meta-analysis, and narrative synthesis. Lancet Psychiatry.

[4] Kessler R.C., Foster C.L., Saunders W.B., Stang P.E. (1995). Social consequences of psychiatric disorders, I: Educational attainment. Am. J. Psychiatry.

[5] Kessler R.C., Walters E.E., Forthofer M.S. (1998). The social consequences of psychiatric disorders, III: Probability of marital stability. Am. J. Psychiatry.

[6] Wood L., Alsawy S. (2016). Patient experiences of psychiatric inpatient care: A systematic review of qualitative evidence. J. Psychiatr. Intensive Care.

[7] Johansson I.M., Lundman B. (2002). Patients’ experience of involuntary psychiatric care: Good opportunities and great losses. J. Psychiatr. Ment. Health Nurs..

[8] Shields M.C., Davis K.A. (2024). Inpatient Psychiatric Care in the United States: Former Patients’ Perspectives on Opportunities for Quality Improvement. J. Patient Exp..

[9] Borge L., Fagermoen M.S. (2008). Patients’ core experiences of hospital treatment: Wholeness and self-worth in time and space. J. Ment. Health.

[10] Polacek M.J., Allen D.E., Damin-Moss R.S., Schwartz A.J.A., Sharp D., Shattell M., Souther J., Delaney K.R. (2015). Engagement as an element of safe inpatient psychiatric environments. J. Am. Psychiatr. Nurses Assoc..

[11] Ikai S., Uchida H., Mizuno Y., Tani H., Nagaoka M., Tsunoda K., Mimura M., Suzuki T. (2017). Effects of chair yoga therapy on physical fitness in patients with psychiatric disorders: A 12-week single-blind randomized controlled trial. J. Psychiatr. Res..

[12] Martland R., Onwumere J., Stubbs B., Gaughran F. (2024). A feasibility study of high intensity interval training intervention in inpatient mental health settings. Psychiatry Res. Commun..

[13] Sheaves B., Freeman D., Isham L., McInerney J., Nickless A., Yu L.M., Rek S., Bradley J., Reeve S., Attard C. (2018). Stabilising sleep for patients admitted at acute crisis to a psychiatric hospital (OWLS): An assessor-blind pilot randomised controlled trial. Psychol. Med..

[14] Boden M.T., Gaudiano B.A., Walser R.D., Timko C., Faustman W., Yasmin S., Cronkite R.C., Bonn-Miller M.O., McCarthy J.F. (2016). Feasibility and challenges of inpatient psychotherapy for psychosis: Lessons learned from a Veterans’ health administration pilot randomized controlled trial. BMC Res. Notes.

[15] Tyrberg M.J., Carlbring P., Lundgren T. (2017). Implementation of acceptance and commitment therapy training in a psychiatric ward: Feasibility, lessons learned and potential effectiveness. J. Psychiatr. Intensive Care.

[16] Gaudiano B.A., Ellenberg S., Johnson J.E., Mueser K.T., Miller I.W. (2023). Effectiveness of acceptance and commitment therapy for inpatients with psychosis: Implementation feasibility and acceptability from a pilot randomized controlled trial. Schizophr. Res..

[17] Jacobsen P., Peters E., Robinson E.J., Chadwick P. (2020). Mindfulness-based crisis interventions (MBCI) for psychosis within acute inpatient psychiatric settings: A feasibility randomised controlled trial. BMC Psychiatry.

[18] Davidson L., Hammond V., Maguire T. (2009). The implementation of a ‘Living with Voices’ group in a psychiatric intensive care unit: A pilot study. J. Psychiatr. Intensive Care.

[19] Wood L., Byrne R., Enache G., Morrison A.P. (2018). A brief cognitive therapy intervention for internalised stigma in acute inpatients who experience psychosis: A feasibility randomised controlled trial. Psychiatry Res..

[20] Sharma G., Schlosser L., Jones B.D., Blumberger D.M., Gratzer D., Husain M.O., Mulsant B.H., Rappaport L., Stergiopoulos V., Husain M.I. (2022). Brief app-based cognitive behavioral therapy for anxiety symptoms in psychiatric inpatients: Feasibility randomized controlled trial. JMIR Form. Res..

[21] Fine A.H., Tedeschi P., Morris K.N., Elvolve E., Fine A.H. (2019). Forward thinking: The evolving field of human-animal interactions. Handbook on Animal-Assisted Therapy: Foundations and Guidelines for Animal-Assisted Interventions.

[22] Chandler C.K. (2012). Animal Assisted Therapy in Counseling.

[23] Barker S.B., Dawson K.S. (1998). The effects of animal-assisted therapy on anxiety ratings of hospitalized psychiatric patients. Psychiatr. Serv..

[24] Barker S.B., Pandurangi A.K., Best A.M. (2003). Effects of animal-assisted therapy on patients’ anxiety, fear, and depression before ECT. J. ECT.

[25] Nathans-Barel I., Feldman P., Berger B., Modai I., Silver H. (2004). Animal-Assisted Therapy Ameliorates Anhedonia in Schizophrenia Patients: A Controlled Pilot Study. Psychother. Psychosom..

[26] Kamioka H., Okada S., Tsutani K., Park H., Okuizumi H., Handa S., Oshio T., Park S.J., Kitayuguchi J., Abe T. (2014). Effectiveness of animal-assisted therapy: A systematic review of randomized controlled trials. Complement. Ther. Med..

[27] Lee H., Herbert R.D., Lamb S.E., Moseley A.M., McAuley J.H. (2019). Investigating causal mechanisms in randomised controlled trials. Trials.

[28] Lang U.E., Jansen J.B., Wertenauer F., Gallinat J., Rapp M.A. (2010). Reduced anxiety during dog assisted interviews in acute schizophrenic patients. Eur. J. Integr. Med..

[29] Rodriguez K.E., Green F.L., Binfet J.T., Townsend L., Gee N.R. (2023). Complexities and considerations in conducting animal-assisted intervention research: A discussion of randomized controlled trials. Hum.-Anim. Interact..

[30] Finistrella M., Flores P., Frediani G. (2024). Animal-assisted therapy in patients affected by schizophrenia and schizophrenic-related disorders: A scoping review. Adv. Med. Psychol. Public Health.

[31] Evlat G., Wood L., Glover N. (2021). A systematic review of the implementation of psychological therapies in acute mental health inpatient settings. Clin. Psychol. Psychother..

[32] Teresi J.A., Yu X., Stewart A.L., Hays R.D. (2022). Guidelines for designing and evaluating feasibility studies. Med. Care.

[33] Eldridge S.M., Chan C.L., Campbell M.J., Bond C.M., Hopewell S., Thabane L., Lancaster G.A. (2016). CONSORT 2010 statement: Extension to randomised pilot and feasibility trials. BMJ.

[34] Gee N.R., Townsend L., Friedmann E., Barker S., Thakre T.P., Mueller M. (2025). A pilot randomized controlled trial to examine the impact of a therapy dog intervention on loneliness in adult patients hospitalized in a psychiatric unit. Front. Psychiatry.

[35] Barker S.B., Vokes R.A., Barker R.T. (2019). Animal-Assisted Interventions in Health Care Settings: A Best Practices Model for Establishing New Programs.

[36] Julious S.A. (2005). Sample size of 12 per group rule of thumb for a pilot study. Pharm. Stat..

[37] Chodosh J., Orlando Edelen M., Buchanan J.L., Yosef J.A., Ouslander J.G., Berlowitz D.R., Streim J.E., Saliba D. (2008). Nursing home assessment of cognitive impairment: Development and testing of a brief instrument of mental status. J. Am. Geriatr. Soc..

[38] Russell D.W. (1996). UCLA Loneliness Scale (Version 3): Reliability, validity, and factor structure. J. Pers. Assess..

[39] Wongpakaran N., Wongpakaran T., Pinyopornpanish M., Simcharoen S., Suradom C., Varnado P., Kuntawong P. (2020). Development and validation of a 6-item revised UCLA Loneliness Scale (RULS-6) using Rasch analysis. Br. J. Health Psychol..

[40] McComb S.E., Goldberg J.O., Flett G.L., Rose A.L. (2020). The double jeopardy of feeling lonely and unimportant: State and trait loneliness and feelings and fears of not mattering. Front. Psychol..

[41] Butterworth P., Leach L.S., Pirkis J., Kelaher M. (2012). Poor mental health influences risk and duration of unemployment: A prospective study. Soc. Psychiatry Psychiatr. Epidemiol..

[42] Franke A.G., Schmidt P., Neumann S. (2024). Association Between Unemployment and Mental Disorders: A Narrative Update of the Literature. Int. J. Env. Res. Public Health.

[43] Westermann K., Woud M.L., Cwik J.C., Graz C., Nyhuis P.W., Margraf J., Blackwell S.E. (2021). Feasibility of computerised positive mental imagery training as a treatment adjunct in in-patient mental health settings: Randomised controlled trial. BJPsych Open.

[44] Townsend L., Heatwole J.K., Gee N.R. (2022). Reactivation of a Hospital-Based Therapy Dog Visitation Program during the COVID-19 Pandemic. Animals.

[45] Centers for Disease Control and Prevention Animals and COVID-19. https://www.cdc.gov/coronavirus/2019-ncov/daily-life-coping/animals.html.

[46] American Veterinary Medical Association Animal Assisted Interventions: Guidelines. https://www.avma.org/resources-tools/avma-policies/animal-assisted-interventions-guidelines.

[47] Townsend L., Towsley N., Gee N.R. (2023). “Dogs on Call”: A Community-Engaged Human Subjects Training with Hospital Based Therapy Dog Teams. J. Empir. Res. Hum. Res. Ethics.

